# Using Atomic Force Microscopy to Measure Thickness of Passive Film on Stainless Steel Immersed in Aqueous Solution

**DOI:** 10.1038/s41598-019-49747-0

**Published:** 2019-09-11

**Authors:** Rongguang Wang, Yunhui Li, Tian Xiao, Li Cong, Yunhan Ling, Zhaoxia Lu, Chiharu Fukushima, Isao Tsuchitori, Mohammed Bazzaoui

**Affiliations:** 1 Department of Mechanical Systems Engineering, Hiroshima Institute of Technology, Hiroshima, Japan; 2 Graduate School of Science and Technology, Hiroshima Institute of Technology, Hiroshima, Japan; 3 Department of Rehabilitation, Hiroshima International University, Higashi-Hiroshima, Japan; 4 School of Materials Science and Engineering, Tsinghua University, Beijing, China; 5 School of Chemistry and Chemical Engineering, Guangxi University, Nanning, China; 6 Laboratoire des Matériaux et Environnement, Faculté des Sciences, Université Ibn Zohr, Agadir, Morocco

**Keywords:** Electrochemistry, Techniques and instrumentation

## Abstract

Employing atomic force microscopy (AFM) to measure passive film thickness on stainless steel (SS) in aqueous solution is proposed. SUS304 austenite and SUS329J4L duplex SS samples partly covered by gold were set in a minicell. To remove the original film, the SS surface but gold was etched using dilute sulfuric acid. After cleaning, open circuit potential (OCP), and distance from the sample surface to the top of the gold were measured. They were then immersed in either 1.0% NaCl; 5.0% NaCl; or aqueous solution with pH ranging from 1.0 to 10.0 and measured again. Differences between the first and subsequent measures of OCP suggested a passive film had formed in solution with pH ranging from 2.8 to 10.0. Similarly, differences among AFM measures revealed the observed film thickness increased with increase in pH and with decrease in chloride ions. Also, film thickness in water was greater than that in a vacuum. Comparison of AFM measurements of passive film on the austenite and sigma phases in sensitized SUS329J4L duplex SS revealed the film was thinner on the sigma phase containing more chromium. Taken together, these findings suggest the proposed method is applicable for measuring the thickness of passive films in aqueous solution.

## Introduction

Resistance of stainless steel (SS) to corrosion largely depends on the passive property of film occurring naturally or artificially on its surface. The passiveness of a film generally depends on its composition, structure and geometric distribution, including its thickness.

Passive film on SS has been analyzed using various technologies and methods, including TEM^1^, XPS^2^, AES^3^, SIMS^4^, GDS^5^, XAFS^6^, MU-VRS^7^, Raman spectroscopy^8–11^, ellipsometry^11–15^ and potential-modulated reflection spectroscopy (PMRS)^11,15–18^. Passive film contains a chromium-rich inner oxide layer and an iron-chromium hydroxide outer layer with an amorphous stucture^18–25^. Recently, nanocrystals were also found in the film^26^. All of these properties might change with film thickness, necessitating methods to measure passive film thickness precisely.

Most of the above studies were performed in ultra-high vacuum^1–5,18–26^, but the characteristics of passive film change with environment. Therefore, the film should be studied *in situ*, that is, in the environment from which we want the film to protect the substrate. According to the few reports on *in situ* analysis of the composition of passive film, PMRS reveals the ratio of metal elements in only the top 0.5 nm^16–18^ of the film, and Raman spectroscopy is not sufficiently sensitive to determine the composition of a very thin passive film^11^. The *in situ* thickness of passive film on metals was obtained by measurement of its capacitance by coulometry^27^ and optical constants by ellipsometry^12–15,21^. Ellipsometry has become a common method for investigating changes in the thickness of passive film *in situ* under different applied potentials in solutions^15,21^. However, recently, studies have focused on more precise methods to investigate passive film on SS.

Scanning probe microscopy (SPM) including scanning tunneling microscopy (STM) and atomic force microscopy (AFM) have emerged as potential methods to investigate passive films of nanometer order at the metal/environment interface. STM studies have revealed atomic images of the metal surface in solution by applying a small tunneling current between the probe and steel surface^28–30^. However, STM has not been used to obtain cross-sectional views of passive film on SS *in situ*.

AFM in contact or tapping mode can mechanically trace three-dimensionally the surface of a metal immersed in solution without affecting the metal surface or environment. Using AFM, Zhang observed corrosion products and pitting corrosion of SUS304 SS in 3.5% NaCl solution^31^. However, the lateral resolution of AFM is low when observing steep steps, and usually the obtained morphology is affected by the shape of the probe tip. This makes it difficult to obtain cross-sectional views of passive film using AFM *in situ* by current methods. In the present study, the authors compared thickness measurements among freshly formed passive film on SS *in situ* and in a vacuum. The measurements were obtained using a custom designed minicell and AFM with a vertical resolution of ~0.01 nm to precisely measure passive film thickness. This method should be useful for researching corrosion mechanisms and the growth of passive films on SS *in situ*.

## Results

### Open circuit potential and polarization curves

**Figure 1:**
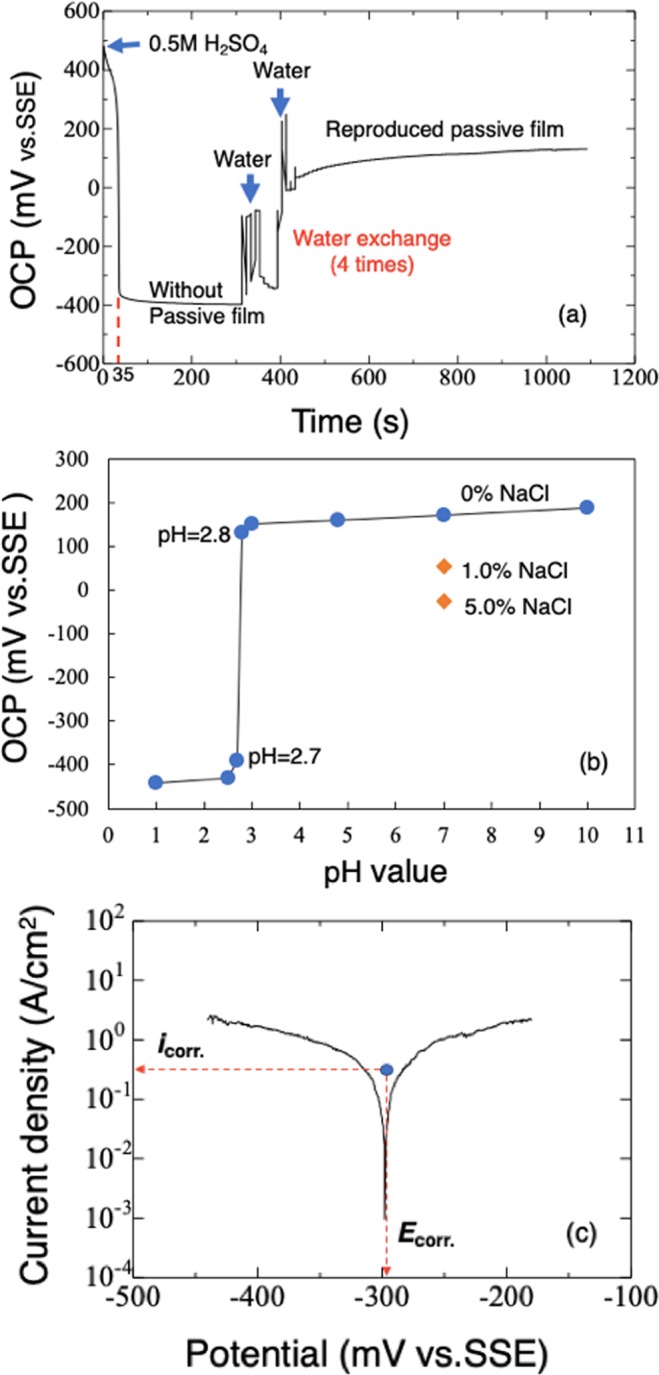
(**a**) OCP variation of SUS304 SS in 0.5 M H_2_SO_4_ solution and in distilled water (pH = 7.0) changed four times in a minicell. (**b**) OCP of SUS304 in aqueous solutions pH = 1.0, 2.5, 2.7, 2.8, 4.8, 7.0 or 10.0. (**c**) A typical polarization curve of SUS304 in 0.5 M H_2_SO_4_ solution open to the air showing the corrosion potential and corrosion current density.

Figure 1(a) shows the open circuit potential (OCP) of SUS304 SS throughout the experiment in the minicell. The OCP sharply decreased to below −350 mV vs. SSE when the sample was immersed in sulfuric acid, indicating the passive film had completely dissolved, exposing bare substrate. After immersion in distilled water for 120 s, having been washed three times with water to remove the acid, OCP increased to 150 mV, indicating formation of a new film. Figure 1(b) shows OCP of SUS304 SS when exposed to aqueous solutions with pH ranging from 1.0 to 10.0 in place of distilled water. OCP did not markedly change when the solution was pH 2.7 or lower, indicating a new passive film could not form. After a marked increase of OCP at pH 2.8, OCP gradually increased with increase in pH. The largest potential was obtained in the solution with pH 10.0. Figure 1(c) shows a polarization curve when the sample was exposed to 0.5 M H_2_SO_4_ solution in the minicell. The dissolution rate was estimated as 0.008 nm/min and 0.013 nm/min, respectively from the Mansfeld and Tafel fitting.

### TEM observation and XPS analysis

**Figure 2:**
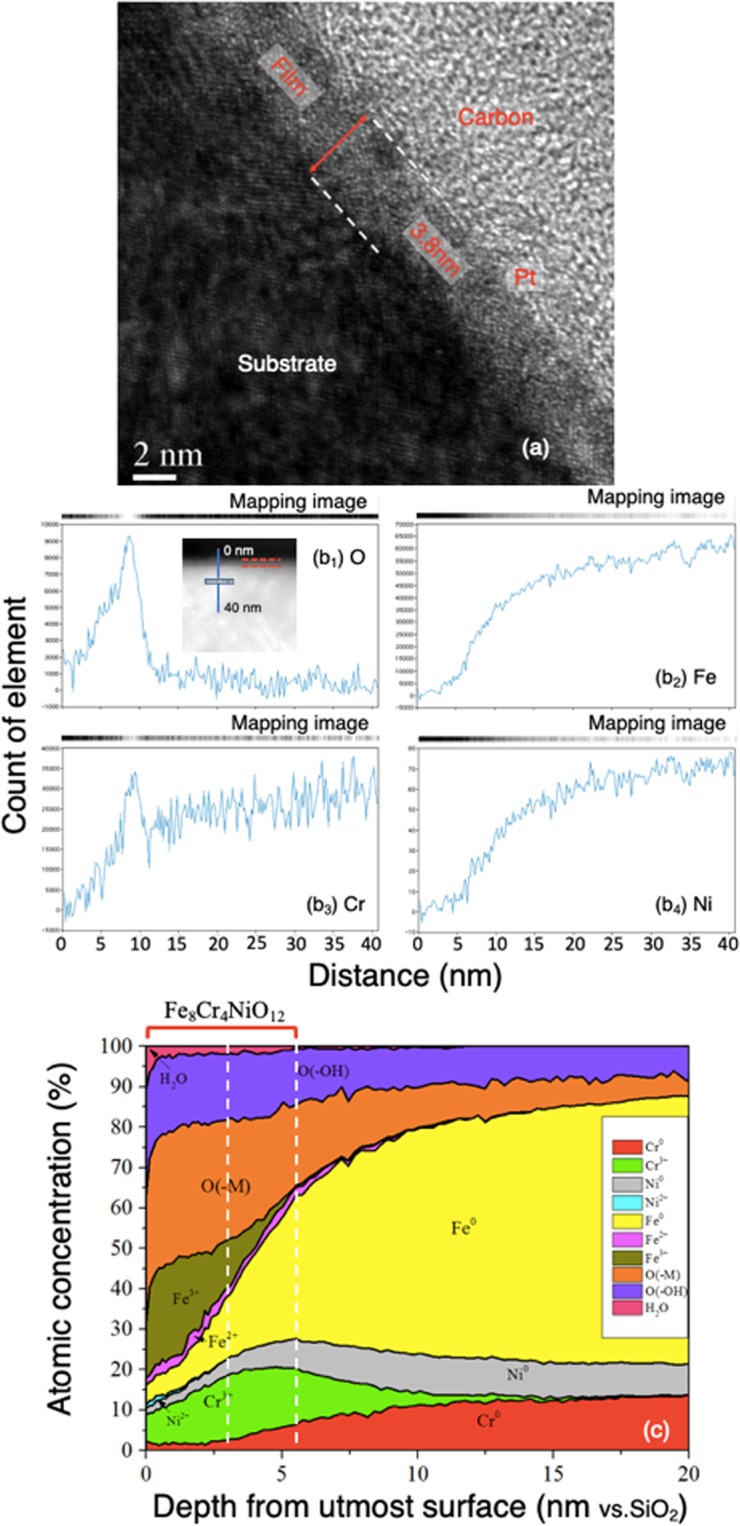
(**a**) Cross section of passive film of SUS304 formed in water (pH = 7.0) as observed by FE-STEM and (**b**) the EELS analysis result. (**c**) The atomic distribution of elements with depth from the topmost surface of the steel analyzed by XPS.

A cross-sectional micro-slice of polished SUS304 SS was prepared as described in ‘TEM and XPS analysis’ in the Methods section. Figure 2(a) shows the cross-section under FE-STEM. The passive film situated between the substrate and the platinum layer would have started to form after wet polishing and continued to form while the sample was cleaned in water and dried in air. It was amorphous and about 3.8 nm thick. From the electron energy-loss spectroscopy (EELS) analysis (Fig. 2(b)), oxygen and chromium were detected in the film with a ratio of Cr to Fe markedly higher in the film than substrate. Figure 2(c) shows the atomic ratios of elements and their distribution depth-wise from the upper surface of the film, as detected by X-ray photoelectron spectroscopy (XPS). The analysis was conducted with intermittent sputter-etching by accelerated argon ions (Ar^+^). The depth in the lateral axis referred to a SiO_2_ film was sputter-etched under the same conditions as the passive film. Therefore, the depth value is proportional but precise. Concentrations of Fe^3+^, Cr^3+^, O^2−^ and OH^−^ were greater than those of Fe°, Ni°, Cr°, Fe^2+^, Ni^2+^ in the outer layer (~3 nm vs. SiO_2_). Presence of adsorbed water was also detected in this layer. In the inner layer (3~6 nm vs. SiO_2_), there were higher concentrations of Fe°, Cr^3+^, O^2−^ and OH^−^ and considerable Cr°, Ni°, but little Fe^2+^, Fe^3+^. The oxides and hydroxides of iron and chromium were enriched in the outer layer, while the oxide and hydroxide of chromium were concentrated in the inner layer. This result corresponds well with previous studies^19–25^. In addition, a little adsorbed water was present in both the outer and the inner layers. Based on the ratios of Fe, Cr, Ni and O atoms, the composition of the passive film is suggested to be Fe_8_Cr_4_NiO_12_.

### *In situ* AFM observations of samples in H_2_SO_4_ solution and water

**Figure 3:**
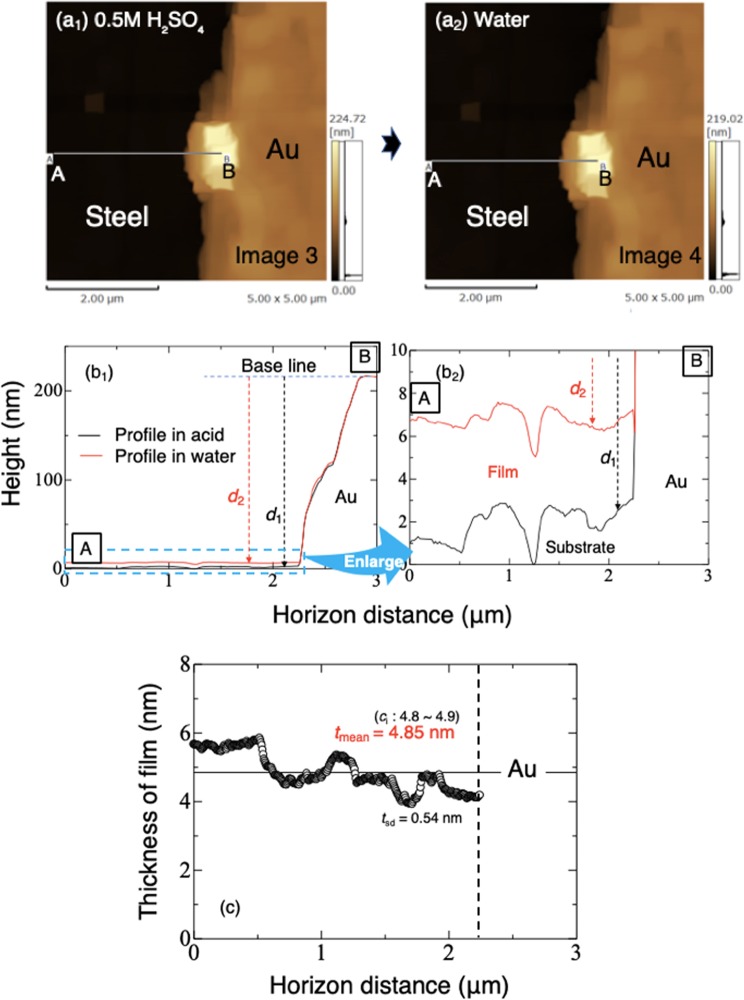
(**a**) Top view images of SUS304 and the gold block in 0.5 M sulfuric acid solution **a**_**1**_ and water (pH = 7.0) **a**_**2**_. (**b**) Two distance profiles obtained from the same positions A-B, in (**a**) for comparison, used to calculate the observed thickness (*t*_o_) of the passive film. (**c**) Observed thickness (*t*_o_) variation of the passive film along A-B line shown in (**a**). *c*i indicates 95% confidence interval of mean thickness.

The 3D morphology of the surface of SUS304 SS with a small gold block on its center was recorded in solutions using AFM. Figure 3(a) shows two top view images of the steel and gold block in 0.5 M sulfuric acid solution (a_1_: after removal of passive film) and after replacement of the acid with water pH = 7.0 (a_2_: after formation of a passive film). The period between recording the two images was about 500 s, during which the acid was replaced with water as described in the Method section. It took 256 s to record each image. In both images, the right side is the gold block, while the left side is the bare steel surface in acid (a_1_) and steel with passive film in water (a_2_). There is no obvious observable difference between them. A line was drawn from point B on the gold block to point A on the steel surface in Fig. 3(a), to obtain two measurement profiles, *d*_1_ and *d*_2_ (Fig. 3(b)). The same positions of the two profiles can be confirmed by comparing the overlapped shapes of the gold block (Fig. 3(b_1_)). In the magnified profiles (Fig. 3(b_2_)), a difference between the distance between *d*_1_ and *d*_2_ in acid and that in water is clear. The zone between *d*_1_ and *d*_2_ can be considered as the *observed shape* of the newly formed passive film. Figure 3(c) shows the variation in thickness (*t*_o_) of the passive film along line A-B shown in Fig. 3(a). The mean thickness is about 4.85 nm, with a standard deviation of 0.54 nm. The mean thickness of the film was confirmed to remain the same after 25 minutes’ immersion in the water. In addition, the thickness fluctuated with position, being slightly decreased near the gold. This may be due a galvanic effect between the gold and SS, which might slightly promote dissolution or instability of the passive film. However, such trend was not found in all samples at different pH. Therefore, the galvanic effect of gold has not been clarified here. The deviation in thickness also probably results from either influence from the roughness, inclusions or grain boundaries. The thickness of the passive film obtained in water (4.85 nm) was larger than that obtained in vacuum (3.8 nm; Fig. 2(a)).

### Observations of samples in solutions with various pH and Cl^−^ concentrations

**Figure 4:**
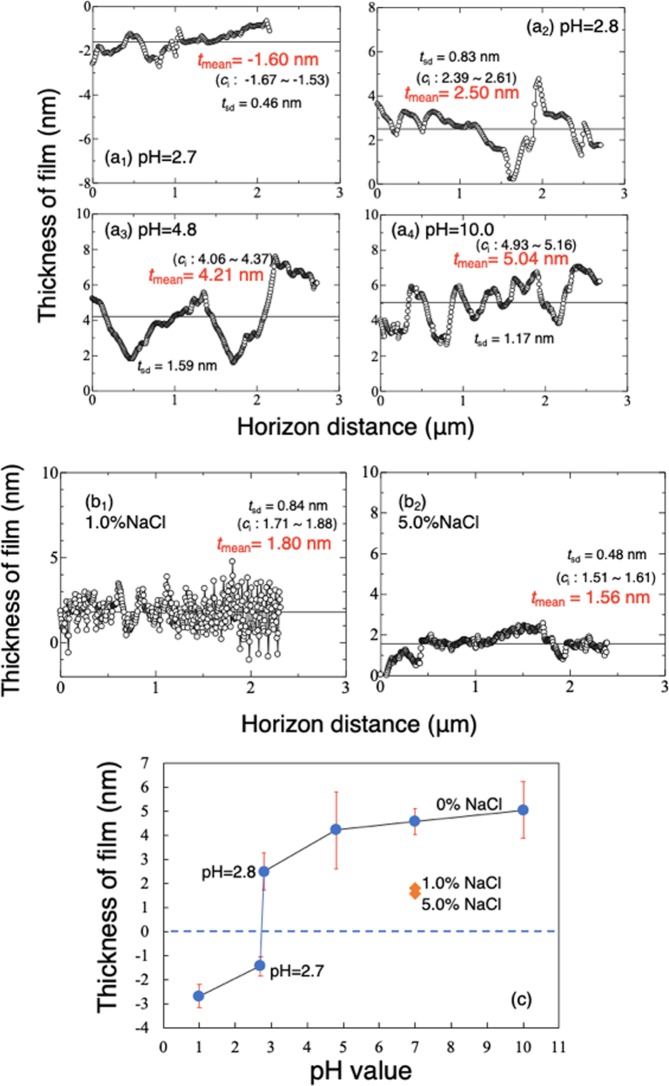
Typical observed thickness (*t*_o_) distributions of passive film in solutions at a range of pH (**a**) and two NaCl concentrations (**b**). (**c**) Mean observed thicknesses (*t*_o_) of passive films formed in the solutions.

Passive films were formed in aqueous solutions with a range of pH and concentrations of NaCl. Typical thickness distributions near the gold block are shown in Fig. 4(a,b). Figure 4(c) shows the mean thickness of passive film formed in the series of solutions. In the solution at pH 2.7, the depth from the gold to the substrate was −1.45 nm, greater than the −2.70 nm obtained in 0.5 M H_2_SO_4_ solution at pH 1.0, indicating dissolution of substrate rather than formation of passive film in these solutions. This corresponds to the OCP measurement (Fig. 1(b)), which shows a much lower value at pH = 2.7, indicating exposure of the bare substrate. As pH increased, passive film formed and became thicker with increase in pH. At pH = 2.8, the thickness was 2.5 nm. The switchover from dissolution to passivation occurred at pH 2.7–2.8, higher than that in solution containing chloride^32^. At pH above 7.0, the thickness remained almost the same. The above results correspond well with the OCP measurements, where the potential restored from -440 mV to much nobler values when the pH was higher than 2.7, and the potential increased with pH. The OCP became obviously noble at pH 2.8 compared to pH 2.7. In Fig. 4(b), the thickness in both 1.0% and 5.0% NaCl solutions (pH = 7.0) is smaller than in solution lacking chloride ions at pH 4.8 to 10.0. This result indicates chloride ions are detrimental to production of a thick film. The Student t-test confirmed the above differences in thicknesses were significant at the *p* = 0.05 level.

## Discussion

In order to understand the variation of the thickness of passive film on the surface of SS observed *in situ*, the following three things should be noted.

1. Replacement of the dilute sulfuric acid with aqueous solutions should be done quickly to minimize substrate dissolution. From the Mansfeld and Tafel fitting (Fig. 1(c)), the lost depth of substrate in 0.5 M H_2_SO_4_ solution during the 120 s it took to wash with water three times and add the final aliquot of water was about 0.016~0.026 nm. In contrast, during this period, corrosion obtained from the *in situ* observation by AFM was about 0.62 nm. Considering that the large dilution effect by the first acid/solution (pH ≧ 2.8) exchange in 30 s can greatly slow down the corrosion, we presumed the corrosion depth during the solution exchange to be 0.2 nm maximum. Therefore, the real thicknesses of the passive film should be a little larger than the figures shown in Fig. 4(c).
2. Whether metal atoms in the film come from the substrate atoms or the dissolved ions near the substrate is important. In the case of the former, the real value of the film thickness would be larger than the value obtained in our study. Sato reported that almost no active dissolution is necessary to produce a passive film on nickel under a constant applied potential in neutral solution if enough active dissolution had already occurred^27,33,34^. In the current study, considering the surface of the steel had dissolved by a depth of 8.0 nm in acid, and that the quick exchange of the solution would not have thoroughly removed the metal ions near the substrate surface, it is quite likely that the passive film would have been able to form without significant further dissolution. Moreover, ellipsometry of passive film on iron in neutral and alkaline solutions showed film formation occurred one second after application of a potential of 800 mV vs. SCE and its thickness became steady at 3.5~4.5 nm after 100 s^35^. In the current study, the film thickness was measured after several hundred seconds from following replacement of sulfuric acid with other solutions, and the measured film thickness was confirmed as stable in water. The authors therefore believed that most of the metal atoms in the passive film came from the solution. However, some would have originated directly from the substrate surface. Therefore, the real thickness of the passive film should be a little thicker than the observed value. According to the above discussion 1 and 2, the thickness obtained by AFM in this work should be more accurately described as “*observed thickness*.”
3. The gold block was used as a thickness reference, because it is chemically and electrochemically stable in sulfuric acid. In Fig. 3(b_1_) and other observations, cross-sectional shape change of the gold block was not evident at the nanometer level. However, due to gold having a nobler potential than SS in solution, there will be a galvanic effect, making the thickness of the adjacent passive film thinner than it would otherwise be. Although this effect was not clearly confirmed in this study, the size of the gold block should be as small as possible.

The thickness of passive film on type 304 steel with applied potentials in solutions at pH 3 and 6.45 measured *in situ* by ellipsometry^36^ was 1.0~2.0 nm at the corrosion potential (*E*_corr._; almost equal to OCP) and increased to a maximum of nearly 4.0 nm with higher potential and higher pH. Almost the same results were obtained on Fe-(5~100)Cr alloy in 1.0 M Na_2_SO_4_ solution at pH 2.0 and 6.0^37^. However, the thickness at *E*_corr._ does not seem to be largely affected by pH^36^. The thickness of passive film on Fe-18~19Cr steels increased from about 1 nm to 2 nm when pH increased from 3 to 6^15,21^. These values are all clearly smaller than those obtained in our study. The reason is not clear, but could be due to the complexity of determining the optical parameters of nanometer film in ellipsometry with special interfaces between the substrate and solution, while the thickness in this study was directly and simply measured by tracing the specimen surface to obtain thickness variation.

**Figure 5:**
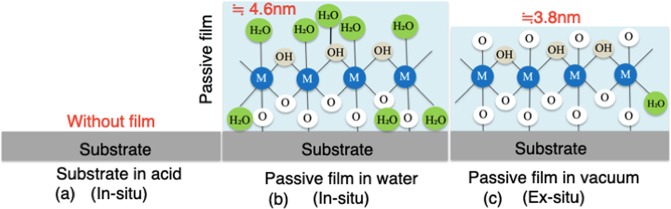
Schematic structures of passive film formed in 0.5 M H_2_SO_4_ solution (**a**) (*in-situ*), in water (**b**) (*in-situ*) and in vacuum (**c**) (*ex-situ*).

Comparing the results obtained by FE-TEM in vacuum (*ex situ*) and by AFM in water (*in situ*), the observed thickness of the passive film in water was larger, especially when taking into account the slight dissolution of the substrate just before the film formation and the thin inner layer of the film at the initial formation stage. The reason is illustrated in Fig. 5. In aqueous solution (a), passive film forms with the hydration of metal atoms, generally following dehydration involving deprotonation^27,38,39^. More water molecules can be contained at potentials below 0.4 V (vs. SCE) in 0.5 M H_2_SO_4_^38^, when a larger number of hydroxy groups and water molecules would be present in the film^38,40,41^. The hydroxy groups bond to metallic ions, while the water molecules exist mainly as little water of crystallization and adsorbed water in the film^27,38^. The water molecules in passive film are thought to play an important role in stabilizing and repairing it^27,38,39,41^. Furthermore, PMRS analysis *in situ* showed much Cr^3+^ was contained in the outmost 0.5 nm layer on Fe-19Cr alloys in solutions at pH 2.0 and 6.0^16^, without giving any information about hydroxy groups. On the other hand, Raman spectroscopy *in situ* revealed the existence of hydroxy groups in films of iron, chromium, and SS in aqueous solutions^9,10^. The water content in passive film on iron was presumed to decrease with decreasing pH until the film became almost anhydrous in solution with pH less than 4.0, while a hydrate outer layer forms in neutral and alkaline solutions^42^. In the current study, when the passive film was transferred from the aqueous solution into an ultra-high vacuum for observation or composition analysis, water molecules would have desorbed, and some hydroxy groups would have left. This would have decreased the thickness of the passive film. Until now, most analyses in previous studies have clarified the structure of passive film *ex situ*, not in solution, showing the existence of considerable hydroxy groups and a small amount of combined water molecules (Fig. 2(c)).

**Figure 6:**
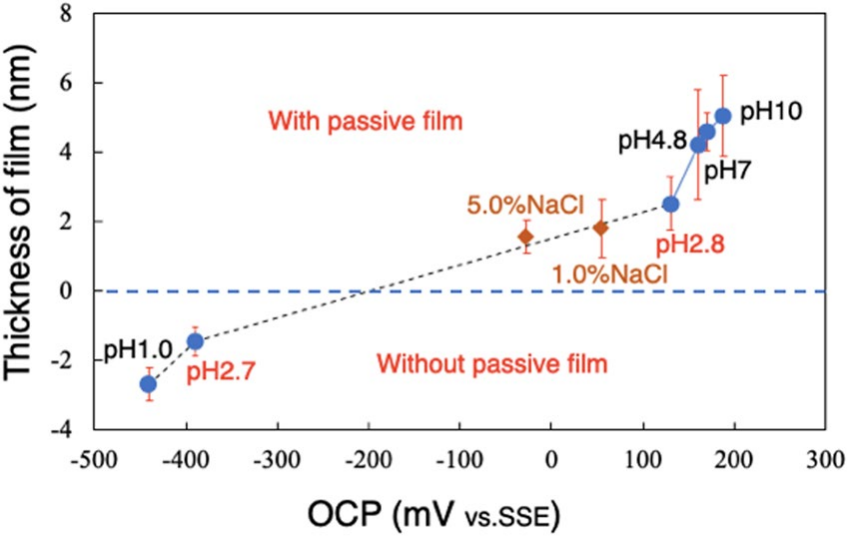
Correlation of the OCP and observed thickness (*t*_o_) of passive film obtained with different solutions.

On the other hand, the thickness of passive film *in situ* increases with increase in pH. More hydroxy groups and water molecules should be incorporated in the film in solutions at higher pH^42^. Figure 6 shows the correlation of the OCP and observed thickness of passive film obtained with different solutions, and shows the same trend as that obtained by ellipsometry on Fe-13~24Cr alloys at applied potentials in solutions with pH 3.0, 6.0 and 6.45^15,21,36^. In the *in situ* PMRS analysis of the top 0.5 nm layer of passive film of Fe-19Cr alloy, the Cr^3+^ ratio decreased with increase in pH from 2.0 to 6.0^16^. With increased applied potential (or OCP), a higher electric field is applied between the solution and steel, which promotes the diffusion of ions, forming a thicker film. On the other hand, the high electronegativity of Cl^−^ ions may have helped them combine with metal ions after they had differentially adsorbed onto the film, which would have impeded film growth.

**Figure 7:**
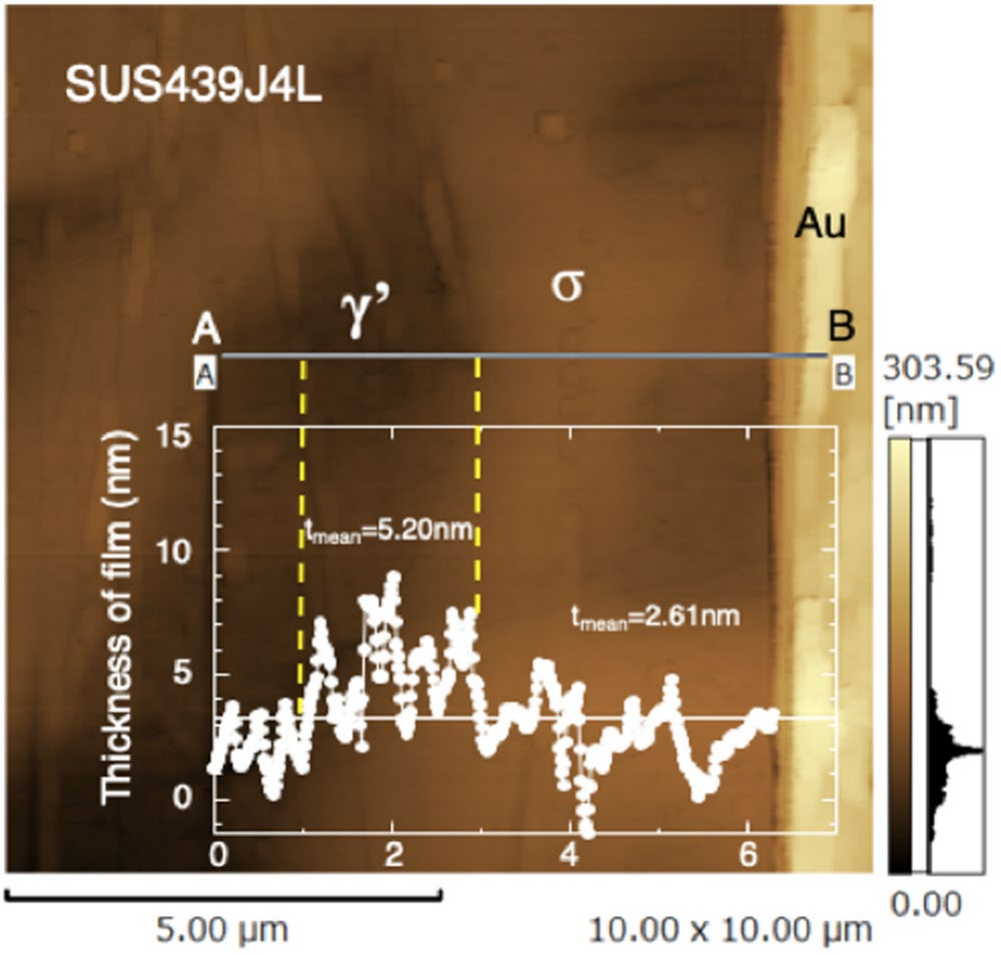
Observed thickness (*t*_o_) of passive film formed on sigma (σ) and gamma prime (γ′) phases of continually cooled (0.02 K/s) SUS439J4L duplex stainless steel immersed in water.

As an application of the above method, Fig. 7 shows the observed thickness of passive film formed in water on the sigma (σ) and gamma prime (γ′) phases in sensitized SUS439J4L duplex SS after continuously cooling at 0.02 K/s between 1073 and 773 K. The solution-treated SUS329J4L generally contains two phases, ferrite (α) and austenite (γ), before sensitization. During the continuous slow cooling, the α phase changes to fine σ phase with high Cr content and γ′ phase with low Cr content to induce prior corrosion around σ phase^43^. In Fig. 7, the passive film on the σ phase was confirmed to be thinner than that on the γ′ phase. This corresponds well to the principle that a stable and thin passive film generally forms on steel with high Cr content^37,44^. The intergranular corrosion of SS occurs preferentially from the Cr-depleted zone around compounds or phases with high Cr content. Therefore, this result is helpful to understand the mechanism of intergranular corrosion in the sensitized SUS439J4L duplex SS, initiated from Cr-depleted zones around the σ phase and then spread to the γ′ phase.

This research provides a new method to investigate *in situ* the thickness of passive film through removal and production of passive film on metals. Therefore, the *in situ* analysis of their real properties is convenient. This method can also be applied to STM^28–31^ or FM-AFM^45^, in which the atomic structure of the top of the passive film might be further clarified. With developments in fast scanning technology of probes and data sampling in the future, it may be possible to elucidate dynamic formation processes of the film. Furthermore, elucidation of passive film on or near different phases, grains, grain boundaries and various inclusions in SS should advance our understanding of corrosion mechanisms and mechanical properties of films in various environments. This may promote the birth of a new of generation SS with much higher corrosion resistance and environment strength.

As a summary, we describe a novel method of measuring *in situ* the thickness of passive film in aqueous solutions by AFM. Original film is first removed by etching in sulfuric acid and then a new film is allowed to form in designated solutions. The removal and production of the passive film can be confirmed by open circuit potential measurements. The observed thickness of the passive film on SUS304 steel in water was 4.85 nm, clearly larger than that observed in vacuum by FE-STEM, which should be attributed to the hydroxy groups and absorbed water molecules in film in water. The observed thickness of the passive film in solution varied with pH. Furthermore, the thickness of the passive film on both the austenite and sigma phases in sensitized SUS329J4L duplex SS was also measured and compared.

## Methods

### Sample preparation

A round plate of SUS304 SS of chemical composition Fe-0.05C-0.50Si-1.11Mn-0.026P-0.003S-18.07Cr-8.04Ni (mass%), which had been solution treated (1303 K × 7.2 ks; N_2_ cooling), was mechanically polished wet by 0.05 μm alumina powder, and then cleaned ultrasonically in water then acetone. Gold was deposited to a thickness of 200 nm in a 1 mm × 1 mm area at the center of the sample’s surface by sputter coating. This was to serve as a thickness reference, as it is inert under all conditions to which it was exposed in this study. SUS329J4L duplex SS of chemical composition Fe-0.029C-0.43Si-0.73Mn-0.038P-0.005S-24.19Cr-5.68Ni-2.68Mo-0.14 N, and annealed by continuously cooling at 0.02 K/s between 1073 and 773 K, with ferrite (α), austenite (γ, γ′) and sigma (σ) phases^43^, was used after being polished and cleaned as above.

### OCP and polarization measurements

OCP and polarization curves of the SU304 steel, without a gold coating, were obtained in a mini electrochemical cell with three electrodes, an SS working electrode, a platinum plate cathode electrode and a reference electrode (SSE: vs. Ag/AgCl). The solutions had a range of pH, and were all at room temperature and exposed to the air. Measurements were made using a potentiostat (CS350, Wuhan Corrtest Instruments Co.). The pH was adjusted by diluted H_2_SO_4_ and NaOH solutions. During polarization measurements in 0.5 M H_2_SO_4_ solution, the potential was swept at a rate of 0.5 mV/s. The potential ranges used in Mansfeld and Tafel fittings were ±15 mV and ±60 mV, respectively.

### TEM and XPS analysis

The polished SUS304 plate was immersed in water for 120 s and then dried in ambient air. A cross-sectional slice measuring 10 μm × 10 μm × 100 nm was machined by focus ion beam (FIB) (FB-2100, Hitachi High-Technologies Co.) after sequential deposition of 2 nm platinum, 100 nm carbon and tungsten. The slice was observed under a field emission scanning electron microscope (FE-STEM) (JEM-3000F, JEOL Ltd.) with EELSE analysis capability. Composition analysis was carried out by X-ray photoelectron spectroscopy (XPS) (Shimadzu Co.: AXIS ULTRA), the X-ray source being the Kα spectrum from a Mg target, generated at 15 kV with an anodic current of 10 mA. The air pressure in the vacuum chamber was lower than 5 × 10^−7^ Pa. The binding energy for each element was scanned at steps of 0.02 eV per 300 ms with intermittent pre-etching by accelerated argon ions (Ar^+^). The components determined from the obtained spectra are shown in Table 1. Their atomic concentrations were calculated using the atomic sensitive factors (ASF). The analysis depth was determined based on the pre-analyzed result of a 20 nm thick SiO_2_ film on a silicon wafer under the same sputter conditions.

**Table 1:** Binding energies of O 1 s, Fe 2p, Cr 2p and Ni 2p spectrum and atomic sensitive factors used for quantitative determination.

Photo peak	Cr 2p3/2		Fe 2p3/2			Ni 2p3/2		O 1 s		
ASF*	2.491		2.947			3.845		0.736		
Component	Cr^0^	Cr^3+^	Fe^0^	Fe^2+^	Fe^3+^	Ni^0^	Ni^2+^	O (-M)	O (-OH)	O (H_2_O)
Binding energy (eV)	575.17	578.01	708.88	711.20	712.91	853.68	586.86	530.08	531.55	532.74

*ASF: Atomic sensitive factor.

### AFM observations

**Figure 8:**
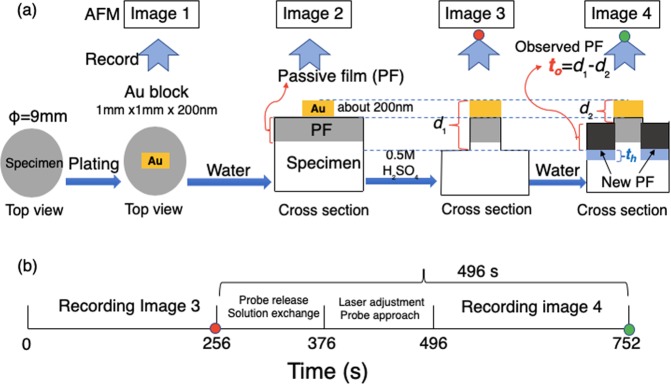
(**a**) Process flow to get the observed thickness (*t*_o_) of passive film formed on stainless steel in water as well as in designated solutions. (**b**) Time period at each stage for capturing images 3 and 4.

The observation was carried out in the above mini electrochemical cell using an atomic force microscopy (SPM-9700, Shimadzu Co.). A microprobe (OMCL-TR800PSA, Olympus Co.) with a tip curvature less than 15 nm was used to trace the specimen surface in contact mode at room temperature. The pressing force between the probe and specimen was kept at 0.13 nN during all observations. Figure 8 illustrates the process used to obtain passive film on the specimen in water (as well as other designated solutions with different pH) and measure it.

The thickness distribution adjacent to the gold block was measured with the specimen exposed to air (image 1), and then deionized water was admitted into the cell. The passive film in water was observed (image 2), then water was replaced by 0.5 M H_2_SO_4_ solution. The specimen was left until a surface layer about 8.0 nm thick had dissolved (image 3), and bare steel was exposed. The distance between the exposed steel to the top of the gold block was recorded as *d*_1_. Over a period of 120 s, the specimen was then washed three times *in situ* with deionized water and then exposed to a fourth and final aliquot of water for a further 120 s with laser adjustment then probe approach. The distance of the new film was then recorded as *d*_2_ (image 4). Image 4 was compared with image 3, the surface shape almost did not change at the nanometer level compared with the subsequent observation. The *observed thickness, t*_o,_ of the new passive film (*t*_o_) in water was calculated as *t*_o_ = *d*_1_ − *d*_2_. The thickness was compared with that obtained in ultra-high vacuum by FE-STEM. Note that the lower part of the passive film (*t*_h_) adjacent to the substrate to the SS surface obtained in image 3 was not taken into account in *t*_o_ due to the reason described in the discussion section.

## Data Availability

The data that support the findings of this study are available from the corresponding author upon reasonable request.
